# Thyroid autoimmunity following alemtuzumab treatment in multiple sclerosis patients: a prospective study

**DOI:** 10.1007/s10238-022-00981-3

**Published:** 2023-01-15

**Authors:** Paraskevi Kazakou, Dimitrios Tzanetakos, Aigli G. Vakrakou, John S. Tzartos, Μaria-Eleptheria Evangelopoulos, Maria Anagnostouli, Panos Stathopoulos, Georgia N. Kassi, Leonidas Stefanis, Constantinos Kilidireas, Evangelia Zapanti

**Affiliations:** 1 Endocrine Unit and Diabetes Centre, Department of Clinical Therapeutics, Alexandra Hospital, School of Medicine, National and Kapodistrian University of Athens, Athens, Greece; 2 Multiple Sclerosis & Demyelinating Diseases Unit, 1st Department of Neurology, Eginition Hospital, School of Medicine, National and Kapodistrian University of Athens, Athens, Greece; 3 Second Department of Neurology, School of Medicine, National and Kapodistrian University of Athens, “Attikon” University Hospital, Athens, Greece; 4 Department of Endocrinology, Alexandra Hospital, Athens, Greece; 5 1st Department of Neurology, Eginition Hospital, School of Medicine, National and Kapodistrian University of Athens, Athens, Greece

**Keywords:** Autoimmune thyroid disease, Alemtuzumab, Multiple sclerosis

## Abstract

Autoimmune thyroid disease (AITD) is the most common adverse effect in alemtuzumab (ALZ) treated relapsing–remitting (RR) multiple sclerosis (MS) patients**.** The objective of this prospective study was to analyze the occurrence, timing of onset, clinical course, and laboratory characteristics of AITD post-ALZ. We evaluated 35 RRMS patients treated with ALZ at a single academic MS center; clinical and laboratory data were collected before ALZ initiation and thereafter quarterly on follow-up with a median of 43.5 months. Seventeen out of 31 patients (54.8%) with no prior history of thyroid dysfunction developed AITD with a mean onset of 19.4 months ± 10.2 (SD) after the first ALZ cycle; Graves’ disease (GD) (*n* = 9); hypothyroidism with positive stimulating thyrotropin receptor antibodies (TRAb) (*n* = 1); Hashimoto thyroiditis (HT) (*n* = 6); HT with hypothyroidism (*n* = 1). Interestingly, seven of nine (77.7%) GD patients showed a fluctuating course. Three out of four patients with preexisting thyroid disease remained stable, whereas one with prior HT and hypothyroidism developed fluctuating GD. All patients with GD commenced antithyroid drugs (ATDs); five continued on “block and replace” treatment; one required radioactive iodine, and one total thyroidectomy. Our analysis showed earlier onset of ALZ-induced AITD in comparison to most other ALZ cohorts; overall, these patients required complex therapeutic approaches of the AITD. We observed a higher rate of fluctuating GD, with earlier onset and lower remission rate than previously reported, which in the majority of patients required prolonged “block and replace” therapy in the minimum dose of each therapeutic agent or more definitive interventions.

## Introduction

ALZ, a humanized anti-CD52 monoclonal antibody, is approved for highly active RRMS, as it has been demonstrated that its administration decreases relapse rate and disability progression either in treatment-naive patients or in patients not responding to first-line immunomodulatory treatments [1]. CD52 is a cell surface marker expressed on T and B lymphocytes, natural killer cells, dendritic cells, and on most monocytes, but importantly not on hematopoietic precursors [2]. ALZ exerts its immunomodulatory action through binding to the CD52 antigen and the subsequent lysis and depletion of mature circulating CD52 + immune cells; thus, a transient but profound immunosuppression status with relatively brief B cell lymphopenia and prolonged T-cell lymphopenia, overlaps and is followed by an immune reconstitution phase. The prolonged modulation of lymphocyte composition and the long-lasting shift of the immunological balance seems to be relevant to the efficacy of this drug [3].

Alemtuzumab in MS is administered intravenously in two treatment cycles, 12 months apart; the first cycle includes 12 mg/day for five consecutive days, and the second, the same dose for three consecutive days; additional cycles may follow if needed. Apart from the infusion-associated reactions and mild to moderate infections, the principal adverse effect of ALZ is the development of secondary autoimmune disorders that can be observed mainly during the immune reconstitution period post-ALZ. The most common secondary autoimmune phenomenon is AITD.

In the general population AITD has been reported to occur in approximately 1–11%. In the Whickham survey, for example, 5% of women and 1% of men had both positive anti-thyroglobulin (anti-Tg) and anti-thyroperoxidase (anti-TPO) antibody tests and a serum thyroid stimulating hormone (TSH) value > 6 [4]. In the NHANES survey, anti-Tg antibodies were positive in 10.4% and anti‐TPO antibodies in 11.3% of the population [5] GD occurs more often in women and has a general population prevalence of 1–1.5%. Approximately 3% of women and 0.5% of men develop GD during their lifetime [6]. In the context of ALZ treatment of MS, AITD has been reported in approximately one-third of treated patients with no previous history of thyroid disorders, with most studies reporting a prevalence between 29.2% and 45.5% [7–13]. In more detail, GD is the most common thyroid disorder (56–71%), followed by HT with hypothyroidism (6–34%), silent thyroiditis (ST) (3–12%), and hypothyroidism with positive TRAb (2–12.5%) [7, 8, 10–12, 14–16]. Some reports suggest that GD occurring in this context may be less aggressive than the non-ALZ-related-GD, while others indicate a more aggressive course with fluctuations between hypothyroidism and hyperthyroidism [7, 8, 12, 14, 15, 17]. The majority of ALZ-treated subjects are reported to develop thyroid dysfunction within 5 years, with a peak incidence in the third year from the first course [13, 18]. A threefold increased risk of AITD by smoking and a sevenfold increased risk by positive family history of thyroid dysfunction have also been reported, whereas the effect of gender is uncertain [12, 17]. Moreover, the presence of high baseline anti-TPO antibodies is associated with a greater risk of developing AITD[16, 19] Other secondary autoimmune conditions such as immune thrombocytopenic purpura (1–3%), Goodpasture syndrome or single cases of autoimmune neutropenia [20], hemolytic anemia [21] and type 1 diabetes have been rarely reported as well [16].

The exact pathogenic mechanism underlying post-ALZ AITD remains unclear; it is considered to be part of an immune reconstitution syndrome, an autoimmune phenomenon characterized by the recovery of the immune cells after lymphopenia. Similar manifestations have also been reported after highly active antiretroviral therapy (HAART) in human immunodeficiency virus (HIV) patients and after allogeneic or autologous hematopoietic stem cell transplantation (HSCT) [13, 22]. Interestingly, post-ALZ AITD has been described rarely or in very limited numbers when ALZ is administered for indications other than MS (e.g., vasculitis, Bechet’s disease, rheumatoid arthritis, neoplasms of the blood) [13]. In contrast, the prevalence of post-ALZ AITD is very high in MS, suggesting a disease-specific shift in the immunological environment during the reconstitution. Interestingly, no genetic association seems to exist between MS and GD. This high prevalence may be explained by the more rapid reconstitution of B cells without adequate regulatory control by T cells [22, 23]. Potentially as a consequence, a thyroid-specific humoral autoimmune response is induced [24]. It has also been suggested that patients who develop post-ALZ autoimmunity have higher basal levels of IL-21, a cytokine that is instrumental for the help T cells (T helper cells) offer to B cells so that their specificity toward the antigen increases. In T cells, cytokine expression indicates a shift toward a T helper cell 2 (Th2) profile, which could be related to high B cell numbers and IgG4 production [23, 25]. Interestingly, a recent study showed a distinct modulation pattern of serum IgG4 compared to the total IgG during the immune reconstitution after ALZ administration in MS patients, and the patients with high IgG4 levels were more prone to autoimmune manifestations; in addition, the highest IgG4 levels were found among patients developing GD[26].

Notably, real-world evidence studies are very few for ALZ, and real-life data concerning ALZ-induced AITD are lacking. In our study, we describe a cohort of RRMS patients who received ALZ courses at a tertiary academic center in Greece, aiming to define the laboratory characteristics, the timing of onset and the course of ALZ-induced AITD. Furthermore, herein we report two cases of successful pregnancies in ALZ-treated patients; one with GD and the other with HT and hypothyroidism.

## Subjects and methods

We included 35 patients with a diagnosis of RRMS that were treated with ALZ at the Multiple Sclerosis and Demyelinating Diseases Unit of “Eginition” University Hospital from January 2016 to October 2021. In the following, all patients were referred to the tertiary Endocrinology Center at “Alexandra” University Hospital for evaluation and monitoring for possible AITD on follow-up. All patient data were collected prospectively. ALZ was administered in two cycles as described above; however, in one patient a third cycle of ALZ (12 mg/day × 3 days) was administered due to MS disease activity 27 months after the second cycle.

Evaluation included data collection of (1) patient demographic details, (2) previous disease-modifying treatments (DMTs) (3) baseline and quarterly (every three months) thyroid function tests including TSH, free thyroxine (fT4), triiodothyronine (T3), anti-TPO antibodies, anti-Tg antibodies and stimulating TRAb, (4) family history of thyroid autoimmunity and (5) smoking status history (current/ever/never), since the latter two parameters are reported to be risk factors for AITD [12, 17]. At baseline and during the quarterly follow-up a clinical review for thyroid-related symptoms was also performed. The primary outcome measure of our study was the number of patients developing AITD post-ALZ. Details of the time of onset of thyroid autoimmunity regarding treatment timeline, condition type, and patient outcomes were recorded.

### Definition of thyroid dysfunction/AITD

The time of onset of thyroid dysfunction/AITD was defined as the first abnormality in thyroid function tests or the presence of thyroid autoantibodies. Hyperthyroidism was defined as low TSH with or without raised fT4/T3 levels; hypothyroidism was defined as elevated TSH with or without low fT4/T3 levels. TRAb-positive hypothyroidism was defined as elevated TSH with positive TRAb (with or without the presence of anti-TPO and anti-Tg antibodies). GD was defined as hyperthyroidism with positive TRAb and/or increased tracer uptake (> 1.5%) on technetium scan or increased vascularity on ultrasound. Fluctuating GD was defined as GD with multiple alternate phases of hyperthyroidism and hypothyroidism, not explained by overtreatment or poor treatment compliance. HT was defined by positive anti-TPO and/or anti-Tg antibodies and negative TRAb, along with characteristic ultrasound findings. ST was defined as thyrotoxicosis followed by spontaneous euthyroidism or hypothyroidism, with negative TRAb, with or without anti-TPO/anti-Tg antibodies and/or reduced or absent tracer uptake on technetium scan [7, 27, 28]. Of note, all patients were evaluated for the presence of endocrine ophthalmopathy using the Clinical Activity Score (CAS) [29].

### Laboratory measurements

Serum TSH, anti-Tg antibodies, and total T3 were measured using automated chemiluminescent immunoassay systems (Immulite 2000 Siemens). Anti-TPO antibodies and fT4 were measured using chemiluminescent immunoassay systems (Roche Cobas Elecsys 2010). Stimulating TRAbs were detected by the IMMULITE^®^ 2000/2000 XPi TSI assay (Siemens). The cutoff for normal TRAb values was < 0.1 U/ml. It should be noted that the upper, accurately quantifiable, limit of this assay is 40 IU/L (with levels greater than this being reported as > 40 IU/L), and therefore we have used the value 40 for calculations related to the present study whenever TRAb levels of > 40 were reported.

### Statistical analysis

Statistical analysis and graphs were performed using GraphPad Prism (Prism 9; GraphPad Software Inc., San Diego, USA). To compare patient TRAb serum levels at different timepoints (baseline and follow-up), the Kruskal–Wallis test for multiple group comparisons including the Dunn posttest was used. Moreover, multiple linear regression models were applied with autoimmunity emergence (in months) as the dependent variable and sex, age at first ALZ administration (in years), smoking, previous DMTs history, and family history of thyroid autoimmunity as independent variables. A two-tailed *p*-value < 0.05 was considered to be statistically significant.

## Results

### Patient characteristics

The baseline characteristics of the 35 patients included in our study are summarized in Table 1. The mean age at the 1st ALZ course was 35.01 years (range, 19–60 years), with a preponderance of female patients [*n* = 31 (89%)]. Thirty-four patients (97%) received the standard regimen of two cycles of ALZ, 12 months apart, while only one patient received a 3rd ALZ cycle due to MS activity. The median disease (RRMS) duration at the 1st ALZ course was 87 months (range, 3–281 months) and the median follow-up period after ALZ initiation was 43.5 months (range, 12–69.5 months). Thirty-three out of 35 patients had received disease-modifying therapies before ALZ [INF-β (*n* = 16), fingolimod (*n* = 16), natalizumab (*n* = 15), glatiramer acetate (*n* = 9), dimethyl fumarate (*n* = 2), and teriflunomide (*n* = 2), whereas two patients were disease-modifying therapy-naive.

**Table 1:** Patient demographics and nature of AITD

	All patients	Patients with new onset AITD (A)	Patients without new onset AITD (B)	Statistical difference; A vs. B
Number	35	17	18	–
Sex	–	–	–	–
Male, *n* (%)	4 (11%)	3 (17,65%)	1 (5,56%)	NS
Female, *n* (%)	31(89%)	14 (82,35%)	17 (94,44%)	NS
Age at first ALZ course, years, mean ± SD (range)	35.0 ± 8.1 (19–60.1)	35.97 ± 9.6 (23–60.1)	34.11 ± 6.4 (19–50.8)	NS
MS disease duration at first ALZ course, months, median (range)	87 (0.8–281)	74 (0.8–216)	97 (3–281)	NS
Follow-up from the first ALZ administration, months, median (range)	43.5 (12–69.5)	35.47 (12–67.5)	45.47 (29.5–69.5)	0.0116
Number of ALZ courses	–	–	–	–
2 courses	34	16	18	NS
3 courses	1	1	0	NS
Interval to onset of AITD from the 1st ALZ course, months, mean ± SD (range)	19.4 ± 10.2 (2.9 – 35.6)	19.4 ± 10.2 (2.9 – 35.6)	N/A	N/A
Type of AITD, n (%)	–	–	N/A	N/A
GD	9 (25.7)	9 (52.9)	N/A	N/A
Hypothyroidism with positive TRAb	1 (2.9)	1 (5.9)	N/A	N/A
HT	6 (17.1)	6 (35.3)	N/A	N/A
HT with hypothyroidism	1 (2.9)	1 (5.9)	N/A	N/A

*MS* Multiple sclerosis; *AITD* Autoimmune thyroid disease; *ALZ* Alemtuzumab; *GD* Graves’ disease; *TRAb* Thyrotropin receptor antibodies, *HT* Hashimoto thyroiditis; *Ν/Α* Not applicable

Anti-TPO antibodies, anti-Tg antibodies and stimulating TRAb were tested prior to ALZ initiation in all patients. TRAbs were negative in all patients. Four out of 35 patients had preexisting thyroid disease. In more detail, three patients had positive anti-TPO and/or anti-Tg antibodies prior to ALZ initiation; of those, two were under treatment with levothyroxine due to hypothyroidism and one had toxic multinodular goiter (MNG) with subclinical hyperthyroidism. One additional patient had hypothyroidism under treatment with levothyroxine without positive anti-TPO/anti-Tg antibodies.

### Clinical/laboratory features of AITD

During the follow-up, 54.8% of patients without previous history of TD (17 out of 31; 14 female and 3 male) developed AITD. The mean time from the first ALZ administration to AITD onset was 19.4 months ± 10.2 (mean ± SD); range, 2.9–35.6 months. Seven patients developed AITD after the first ALZ course (mean ± SD 10.3 ± 4.7 months), and 10 additional patients developed AITD after the second ALZ course (mean ± SD 13.5 ± 7.6 months). The incidence of thyroid dysfunction and during the first year of follow-up was 23.3% (seven new cases out of 31), and 41.31% after the second ALZ course and during the second year of follow-up (ten new cases of the remaining 24).

More specifically, nine out of 17 patients (52.9%) developed GD; one patient (5.9%) exhibited hypothyroidism with positive stimulating TRAb; six patients (35.3%) developed HT; and one (5.9%) HT with hypothyroidism. The mean time to anti-TPO and/or anti-Tg antibody positivity onset (from first ALZ infusion) in patients with HT ± hypothyroidism was 19.2 ± 12.7 months (mean ± SD). No cases of ST were recorded (Table 1).

### GD and TRAb-positive hypothyroidism

TRAbs were detected in 10 patients (mean ± SD TRAb level, 20.7 ± 16.5 U/L). The mean time to TRAb positivity onset from the first ALZ infusion was 23.5 ± 7.4 months (mean ± SD). The median follow-up since the onset of TRAbs was 24.9 months (range, 16.5–57.2). Nine out of 10 TRAb-positive patients manifested GD. In six of nine patients who exhibited GD, anti-Tg with or without anti-TPO antibody positivity occurred a few months before the occurrence of TRAb. Seven out of nine GD patients (77.7%) exhibited fluctuating thyroid status, transitioning from hyperthyroidism to hypothyroidism and vice versa; the remaining two patients had a “conventional” course of GD. By “conventional” we refer to the typical, not fluctuating course, described in patients of the general population.

Of specific interest, one patient developed hypothyroidism associated with surprisingly high stimulating TRAb levels (> 40 U/L; reference range (rr) < 0.1), as well as anti-Tg antibodies (> 4000 U/ml; rr < 115), while anti-TPO antibodies were borderline positive (35.8 U/ml; rr < 34).

**Fig. 1:**
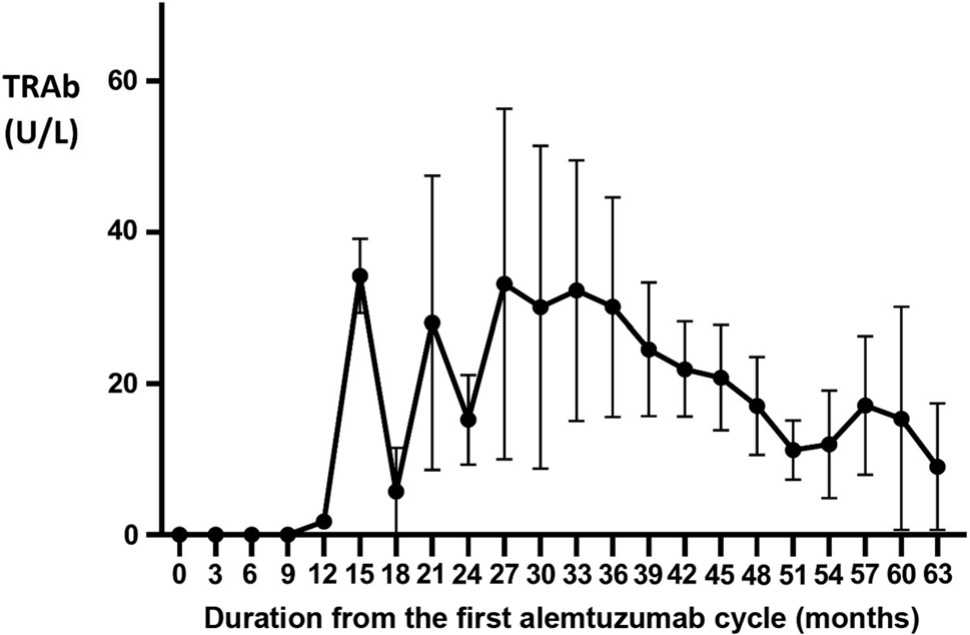
Time course of TRAb in all MS patients treated with alemtuzumab (*n* = 10) with GD and hypothyroidism with positive TRAb. To compare TRAb serum levels in our patients during several months after treatment initiation the Kruskal–Wallis test was used. Dots represent the mean and bars the standard error of the mean (SEM). (*P*-value = 0.001)

Figure 1 shows the time course of mean TRAb levels in all 10 TRAb-positive patients. As depicted, the mean TRAb level begins to rise nine months after the 1st ALZ course and reaches the first peak 15 months post-1st ALZ treatment (also 3 months after the 2nd ALZ treatment); the first peak is followed by a nadir at 18 months, with subsequent lower peaks and nadirs reflecting the fluctuating course of most cases. Later time points reveal a steadier course with a tendency toward a slight decline of mean TRAb levels.

### Treatment modalities and outcomes of AITD

Regarding the therapeutic management of our cohort, all nine patients with GD were initially treated with ATD treatment; four patients with fluctuating GD and one with “conventional” GD continued on “block and replace” treatment with the minimum dose of each therapeutic agent needed (Table 2). One female patient with fluctuating GD on “block and replace” ATD therapy had to undergo total thyroidectomy due to a history of cardiac arrhythmias. Forty months post-surgery, TRAbs were still positive (TRAb 18.1 U/L; (rr) < 0.1); the same patient also developed mild Graves’ ophthalmopathy post-surgery while thyroidectomy revealed a papillary microcarcinoma. Another female patient with fluctuating GD desiring pregnancy was placed on “block and replace” therapy for 41 months, exhibited hyperthyroidism and finally underwent radioactive iodine treatment; interestingly, her TRAb levels were constantly elevated. The remaining GD patients achieved a stable euthyroid state with treatment with ATD ± levothyroxine (up to their last follow-up visit).

**Table 2:** Treatment and outcome of Graves’ disease developing after alemtuzumab therapy

	Fluctuating Graves’ disease	“Conventional” Graves’ disease
Number	7	2
Therapy	–	–
ATD treatment, *n*	3	1
“Block and replace” ATD treatment in the minimum dose of each therapeutic agent, *n*	2	1
“Block and replace” ATD treatment in the minimum dose of each therapeutic agent, plus RAI, *n*	1	0
“Block and replace ATD” treatment in the minimum dose of each therapeutic agent, plus surgery (total thyroidectomy), *n*	1	0
Outcome	–	–
Resolution, *n*	1 (with euthyroidism after ocrelizumab)	0
Graves’ ophthalmopathy, *n*	4	0

*ATD* antithyroid drug; *RAI* radioactive iodine

Remission was reported in one female patient only who was started on therapy with ocrelizumab due to breakthrough MS disease activity. In more detail, this patient developed fluctuating GD and received “block and replace” treatment for 24 months. Due to an MS relapse, the patient was started on ocrelizumab iv infusions with subsequent control of MS activity and concurrent remission of GD with normalization of TRAb levels occurring 12 months post-ocrelizumab initiation. Overall, four patients (44.4%) developed mild Graves’ ophthalmopathy (GO) not requiring further treatment, all of which demonstrated fluctuating GD.

The patient with HT and hypothyroidism, as well as the patient with TRAb-positive hypothyroidism are euthyroid under levothyroxine replacement therapy.

The course of the four patients with thyroid dysfunction prior to ALZ is as follows: The one of the two female patients with HT and hypothyroidism prior to ALZ developed fluctuating GD (TRAb 1,8 U/L; rr < 0,1] 13 months after the first ALZ infusion, requiring treatment with ATD in a dose-reducing regimen on last follow-up. The other female patient with HT and hypothyroidism remained euthyroid under the initial treatment with levothyroxine. The patient with prior hypothyroidism without positive anti-TPO/anti-Tg antibodies did not develop any autoimmunity and remained euthyroid under the initial treatment with levothyroxine. Finally, the female patient with HT and preexisting toxic MNG remained stable with subclinical hyperthyroidism until the last follow-up and is scheduled for surgery.

### Risk factors and AITD

To assess potential causal correlations and risk factors for AITD, we performed multiple linear regression analyses using age at the time of ALZ treatment, sex, smoking status, previous DMTs, positive baseline anti-TPO/anti-Tg antibodies and family history of AITD as independent variables; however, no significant relationship was found. Only in patients who were treated with fingolimod as last treatment before ALZ a trend for an increased incidence of TRAb positivity was reported albeit with no statistical significance.

### Pregnancy outcomes

It is noteworthy that two successful pregnancies were recorded during follow-up. The first, a 32-year-old woman, developed HT with hypothyroidism (TSH 57; rr 0.4–4 μU/ml, fT4 0.58; rr 0.89–1.76 ng/dl) 11 months after the first ALZ course. TRAbs were negative, while anti-TPO and anti-Tg antibodies were highly elevated; > 1300 U/ml (rr < 34) and > 500 U/ml (rr < 40), respectively. She gave birth to a healthy boy 22 months after the second ALZ course. The levothyroxine dose was slightly increased during pregnancy to maintain a euthyroid status. She is currently euthyroid under levothyroxine treatment (75 μg/day) with persisting anti-TPO antibodies (115.3 U/ml; rr < 34) and normal anti-Tg antibodies. The second, a 31-year-old woman, developed GD hyperthyroidism during the fifth week of pregnancy (32 months after the first ALZ course); thyroid function showed low TSH < 0.004 μU/ml, elevated fT4 (57.17 pmol/l; rr 12–22), elevated fT3 (fT3) (20.86 pmol/l; rr 3.1–6.8), and a high TRAb level (2.94 U/L; rr < 0.1). She was commenced on propylthiouracil (PTU) that was stopped at the end of the first trimester as TRAb titer declined to 1.44 U/L (rr < 0.1) and hyperthyroidism resolved. At gestational week (GW) 18, treatment with low-dose carbimazole (5 mg/day) was started because of a relapse of mild hyperthyroidism. At GW25, TRAb titer further declined to 0.859 U/L (rr < 0.1) and carbimazole was discontinued and no ATD treatment was needed until the end of gestation. During the whole pregnancy serial fetal sonograms showed normal development and no sign of fetal thyrotoxicosis. A healthy girl weighing 3460 gr was spontaneously delivered at GW40 without complications. The newborn baby did not develop perinatal thyrotoxicosis. 11 months after delivery, this patient had a recurrence of GD; at the last follow-up, she was well controlled with declining TRAb (0.527 U/l) under “block and replace” treatment.

## Discussion

AITD occurred slightly more frequently post-ALZ treatment in our cohort of MS patients (54.8%) compared to previous studies (29–45%)[7, 8, 10–12, 14, 15, 17, 19, 30]; only Muller et al. [16] have reported a higher rate of 68.8%, although with a longer mean ± SD follow-up of 9 ± 2.5 years. In our cohort the median follow-up was 43.5 months (range, 12–69.5 months), similarly to most previous studies. In more detail, previous studies with a median follow-up of 36 [19], 84 [8], 57.3 [12], and 34 [17] months have reported AITD prevalence of 29.2%, 41%, 34%, and 16.5%, respectively. A metanalysis by Scapaticcio et al. [11] including seven studies with a median follow-up of 24–60 months has reported an estimated prevalence of 33%. Moreover, Pariani et al. [7], in their study with a mean follow-up of 67 months (range 6–251), reported a prevalence of 41.1%. Similarly, Manso et al. [14] in their retrospective study have reported a AITD prevalence of 39% during a mean follow-up period of 32 ± 17 months. Therefore, we report an increased rate of AITD compared to many studies with similar or longer mean follow-up.

Additionally, GD was the most common disorder (52.9%) we noted, in accordance with previous studies [7, 11, 12, 14, 17, 19]. The mean time to AITD onset was 19.4 months from the first ALZ course. Specifically, the mean onset of AITD was 10.2 months after the first ALZ course in seven cases and 13.5 months after the second course in the rest 10 cases, showing a time interval shorter than most previous studies have reported. According to these studies, the incidence peak was 28.5 months from the last ALZ dose and the median time of onset was 32 months after the first course or the 91% of AITD cases occurred within four years from the last ALZ course [7, 8, 11–13, 17]. Only Manso et al. [14] in their real-life retrospective cohort have reported, similarly to our results, a mean time interval to AITD onset of 17 months after the first ALZ course and 10 months after the last. In our cohort, HT ± hypothyroidism was the most frequent thyroid disorder after GD (41.2%), followed by hypothyroidism with positive stimulating TRAb (5.9%), in line with previous studies [7, 11–15]. No ST was recorded, while several reports mentioned a low prevalence of 4–12% [7, 11, 12, 14].

We further recorded a higher rate of patients with fluctuating GD (77.7%) than previous studies (prevalence of 15–67%) [7, 11, 12, 14, 16]. Possibly due to this fluctuation, we reported a very low remission rate of 11%. Permanent remission occurred only in 1 patient with fluctuating GD, who had been on “block and replace” ATD therapy for 24 months, 1 year after ocrelizumab treatment. Remission of GD 1 year after CD20 depletion points to possible involvement B cell deregulation in the pathogenesis of post-ALZ GD and highlights the need for an early switch to B cell depletion (if indicated by MS activity) or mitigation of B/T cell imbalance with low-dose rituximab [31]. On the last follow-up, the remaining GD patients were on ATD therapy ± levothyroxine with positive stimulating TRAb, while one patient needed radical treatment with radioactive iodine and another underwent total thyroidectomy. Similarly, Manso et al. [14] in their cohort recorded a low remission rate (18%) and Pariani et al. [7] recorded a remission rate of 34%. In contrast, initial clinical trials and earlier reports suggest that post-ALZ GD has a more favorable course than the “conventional” GD [8, 12, 17, 32] with a higher rate of response to ATD therapy [23, 33] and higher remission rates of 37–57% [8, 11, 12]. However, our findings are in accordance with a growing body of data indicating that GD after ALZ treatment seems to have a more unpredictable course compared to conventional GD, requiring long-term ATD therapy or possible radical treatment (radioactive iodine or surgery); it should be noted here that “conventional” GD has a remission rate of 50% [7, 12, 14, 34]. As previously reported, the fluctuating phenotype of GD is indicative of the alternating presence and/or coexistence of stimulating and inhibitory TRAb with a resultant predominant stimulation or inhibition of thyroid hormone secretion [7, 14, 16]. This fluctuation from hyperthyroidism to hypothyroidism and vice versa has been described in rare cases after levothyroxine treatment for hypothyroidism or after ATD treatment of conventional GD [35]. Accordingly, the presence of hypothyroidism with high stimulating TRAb titer may suggest the coexistence of a higher titer of blocking TRAb. Of note, the TRAb assay we performed only identifies stimulating TRAbs.

We found no correlation of AITD development with risk factors such as age at the time of ALZ treatment, sex, smoking habits, previous DMTs, positivity of baseline anti-TPO/anti-Tg antibody measurements, or history and family history of AITD. Scappaticcio et al. [11] report in their meta-analysis that data concerning risk factors for the development of post-ALZ AITD are scarce. Some studies have shown that smoking and positive family history of thyroid autoimmunity increase the risk of AITD [12, 17], while other studies report no correlation with sex, age, smoking status, or family history of thyroid dysfunction [14, 19]. However, most studies agree that baseline positivity of anti-TPO/ and or anti-Tg antibodies is significantly associated with increased risk for the development of AITD [12, 14, 16, 19, 30]. Few data concerning previous DMTs as a risk factor for AITD are found in the literature. Nevertheless, our results are in accordance with previous studies [7, 11, 14, 17, 19], which reported no significant association. Only the study by Pfeuffer et al.[36] demonstrated an increased risk of secondary autoimmunity among patients previously treated with fingolimod. In our study a trend for an increased occurrence of TRAbs in patients who received fingolimod as last treatment before ALZ was found as well, perhaps not reaching significance due to the number of patients.

In our cohort, the 17 patients who developed AITD had negative baseline anti-TPO/anti-Tg antibodies. Of note, only one patient with positive baseline anti-TPO antibodies developed fluctuating GD. Our negative results concerning baseline anti-TPO/anti-Tg antibody positivity as a risk factor may be due to the small number of such patients. Overall, 44.4% of our GD patients exhibited mild GO, contrary to previous studies which have shown a rate of 6.25–13.4% [7, 11, 12]. However, during quarterly follow-up, clinical examination in our study included screening for endocrine ophthalmopathy whereas in several other studies, ophthalmopathy screening was not routinely performed and therefore GO may have been underdiagnosed [7].

Interestingly, we also report on two pregnancies post-ALZ treatment; in the first case, the patient developed HT and hypothyroidism, and in the second GD hyperthyroidism. Information concerning incidence and course of post-ALZ AITD and especially ALZ-induced GD during pregnancy is limited to a few reports [37–39] of such challenging cases. Herein, we report a pregnancy, in which GD hyperthyroidism with a high TRAb titer developed within the first trimester and required ATD treatment. Subsequently, hyperthyroidism improved allowing discontinuation of ATD treatment in the third gestational trimester and leading to the delivery of a healthy, euthyroid baby. On the contrary, similar case reports mention a more aggressive GD course with a rise or persistence of the TRAb titer in the third trimester necessitating escalation and continuation of ATD therapy with augmented risk of both fetal and neonatal thyrotoxicosis [37–39]. In the general population, maternal hyperthyroidism occurs in about 0–2.5% of pregnancies [40], and in most cases the cause is GD. As mentioned above, the incidence of AITD is up to 45% of the patients treated with ALZ, of which 60–70% are GD cases [7, 14]. During pregnancy, hyperthyroidism may result in maternal, obstetrical, fetal, and neonatal complications. Fetal and neonatal hyperthyroidism occurs in 1–5% of pregnant mothers with GD, when maternal TRAbs cross the placenta stimulating the fetal thyroid gland, thus leading to excessive thyroid hormone secretion. The risk correlates with the TRAb titer; a maternal TRAb level > 5 IU/L or three times the upper reference range is associated with a higher risk for neonatal hyperthyroidism [41]. In our case, although the TRAb level in the beginning of pregnancy was three times over the upper reference range, hyperthyroidism resolved in the third trimester. In the general population, GD during pregnancy may improve in some patients and allow for the discontinuation of ATD treatment in the third trimester [42], however, in severe cases the risk for fetal and neonatal hyperthyroidism remains high [43]. Concerning future pregnancies, it is important to address the risk for secondary AITD in ALZ-treated women of childbearing age, mainly GD, in conjunction with their treating obstetrician.

The key strength of our study lies in the fact that it is based on real-world clinical and laboratory data prospectively collected by the same multidisciplinary medical team of an academic hospital center, with criteria and goals for treatment remaining constant during the whole study period. Although a limitation compared to other studies might be the lower number of patients, the present study provides real-world data that complement information derived from randomized controlled trials.

In conclusion, post-ALZ AITD may represent a dynamic spectrum of diseases with unique non-classical phenotypes, such as the unpredictable and fluctuating course of GD with resistance to ATD therapy necessitating radioiodine therapy or radical surgery.[7, 11, 12]. Further studies are needed to understand the underlying mechanisms that could be responsible for this immune dysregulation and the pathways related to AITD. Importantly, a detailed, thyroid-specific pre-treatment screening and a thorough long-term follow-up of all ALZ-treated patients can facilitate early diagnosis and effective treatment of possible secondary AITD and should therefore be considered standard of care in this setting. Recent guidelines recommend 3‐monthly thyroid function tests for at least 4 years after the last course of ALZ [13]. According to our results, for the fluctuating course of GD long-term “block and replace” ATD therapy in the minimum dose of each therapeutic agent or definitive treatment (radioiodine or thyroidectomy) may be needed.
